# Depression in Aboriginal men in central Australia: adaptation of the Patient Health Questionnaire 9

**DOI:** 10.1186/1471-244X-13-271

**Published:** 2013-10-20

**Authors:** Alex DH Brown, Ricky Mentha, Kevin G Rowley, Timothy Skinner, Carol Davy, Kerin O’Dea

**Affiliations:** 1 Wardliparingga Aboriginal Research Unit, South Australian Health and Medical Research Institute, PO Box 11060, 5001, Adelaide, South Australia, Australia; 2 Baker IDI (Heart and Diabetes Institute), Alice Springs, PO Box 1294, Northern Territory, Australia; 3 (Onemda VicHealth Koori Health Unit) Melbourne School of Population and Global Health, University of Melbourne, Victoria, Australia; 4 School of Psychological and Clinical Sciences, Charles Darwin University, Darwin, Northern Territory, Australia; 5 School of Population Health, University of South Australia, Adelaide, South Australia

**Keywords:** Indigenous Australians, Depression, Primary health questionnaire 9, Assessment, Mental health

## Abstract

**Background:**

While Indigenous Australians are believed to be at a high risk of psychological illness, few screening instruments have been designed to accurately measure this burden. Rather than simply transposing western labels of symptoms, this paper describes the process by which a screening tool for depression was specifically adapted for use across multiple Indigenous Australian communities.

**Method:**

Potential depression screening instruments were identified and interrogated according to a set of pre-defined criteria. A structured process was then developed which relied on the expertise of five focus groups comprising of members from primary Indigenous language groups in central Australia. First, focus group participants were asked to review and select a screening measure for adaptation. Bi-lingual experts then translated and back translated the language within the selected measure. Focus group participants re-visited the difficult items, explored their meaning and identified potential ways to achieve equivalence of meaning.

**Results:**

All five focus groups independently selected the Primary Health Questionnaire 9, several key conceptual differences were exposed, largely related to the construction of hopelessness. Together with translated versions of each instrument for each of the five languages, a single, simplified English version for use across heterogeneous settings was negotiated. Importantly, the ‘code’ and specific conceptually equivalent words that could be used for other Indigenous language groups were also developed.

**Conclusions:**

The extensive process of adaptation used in this study has demonstrated that within the context of Indigenous Australian communities, across multiple language groups, where English is often a third or fourth language, conceptual and linguistic equivalence of psychological constructs can be negotiated. A validation study is now required to assess the adapted instrument’s potential for measuring the burden of disease across all Indigenous Australian populations.

## Background

Whilst Indigenous Australians experience poorer health than other Australians, there exists little representative data to outline the burden and consequences of mental illness. This is particularly problematic given the national focus on overcoming Indigenous health disadvantage, which will require consideration of the contribution of psychological illness to entrenched health inequalities.

Despite a lack of empirical data, Indigenous Australians are considered to be at particularly high risk of psychological illness [1]: rates of self-harm and suicide are higher [2,3] and national data suggests that Aboriginal people are more likely to be hospitalised for or die from mental and behavioral disorders than their non-Aboriginal counterparts [4]. A systematic review of community surveys of mental illness in Indigenous Australians [5] demonstrated that the prevalence of psychological distress is significantly higher in Indigenous Australians compared to their non-Indigenous counterparts. However, as noted by the authors, these findings should be treated with caution. While the instruments had been validated for use in non-Indigenous populations, the ‘cultural appropriateness’ (p.120) of measures of distress in Indigenous Australian communities remains largely untested.

Few screening instruments have been designed to measure the prevalence of psychological disorders in adult Indigenous Australian populations [6]. A notable exception was a small study [7] that adapted the Patient Health Questionnaire 9 (PHQ-9) [8] for use in a Darwin-based Aboriginal community controlled health service. The modified PHQ-9 tool was administered to 34 Aboriginal and Torres Strait Islander primary care patients diagnosed with ischaemic heart disease. Compared to a semi structured psychiatric diagnostic interview conducted by a medical practitioner (taken as the criterion), the modified PHQ-9 tool demonstrated reasonable sensitivity and specificity but is unlikely to be generalizable for use beyond the local setting. As such, the search for a suitable measure of the overall burden of depression across Indigenous Australian populations continues.

This area is particularly difficult to research. Methodological and conceptual difficulties arise when utilising western systems of illness categorization to study mental illness in cross cultural settings [9]. Depressed individuals can demonstrate a wide range of symptoms, symptoms can be interpreted differently, and methodological barriers exist in the definition and measurement of negative affect [10]. More specifically, depression is expressed linguistically in widely varying ways in different cultural groups [11], particularly as it relates to the translation of the symptoms, antecedents and consequences [12]. Despite the pan-human capacity for sadness and grief, this does not by extension mean that depression as a construct, is universal [13]. Nor should it be assumed that all cultures will consider exactly the same symptoms as valid markers of distress.

Methodologically, it is important to avoid simply transposing or translating western labels of symptoms directly into local, Indigenous ‘labels’. Rather, the collation of lists of population specific terms for various psychiatric or emotional conditions [14] and determination of their conceptual range and meaning is essential. Ultimately it is critical to understand how patients from different cultures experience and express depression [15].

While there are a number of validated screening instruments for the measurement of depression and depressive symptomatology [16,17] there is a need to critically examine inter and intra cultural population differences [18]. Labeling Indigenous people with western diagnostic classifications, without assessment of their equivalence, relevance, acceptability or utility serves little purpose if such labels are devoid of the context and realities of Indigenous people’s lives, their many languages and cultural groups, or the way in which Aboriginal people experience and express psychological distress [19].

This study aimed to develop a robust depression screening instrument which would be both culturally acceptable to and valid for use across Indigenous Australian communities. In addition to demonstrating this face validity with these target populations, the objective was to ensure the utility of the measure [20] in that it was ‘translatable’ by language experts; was brief and able to be self-administered or administered by lay interviewers.

## Methods

### Setting

Alice Springs is a regional town of approximately 30,000 inhabitants situated in the lower half of the Northern Territory of Australia. It is a support hub for the Central Australian region, with a total population of just under 50,000 people spread across approximately 1,000,000 km^2^. Almost 40% of the population identify as Aboriginal people, accounting for 30% of the population within Alice Springs, and 80% of the people living in over 30 discrete remote communities [21].

For many Aboriginal people in Central Australia, English is not their primary language, with many speaking one or more distinct traditional languages in the first instance. These languages are grouped into three main language families - Arandic, Ngarrkic and Western Desert - which contain a number of mutually intelligible/overlapping dialects. For this work, we chose to target the most widely spoken dialects – Pitjantjatjara, Luritja, Pintupi (Western Desert family); Eastern and Central Arrernte and Anmatyerre (Arandic); and Warlpiri (Ngarrkic)^a^.

The study was approved by the Central Australian Human Research Ethics Committee.

### Background qualitative investigations

The adaptation process was informed by detailed qualitative research within the target population. While full details about this qualitative component have previously been published [22], in brief it involved 22 in-depth qualitative interviews that were thematically analysed in order to conceptualise and identify the expression of emotional distress and depression among Aboriginal men.

In brief, depressive symptomatology was common and depression as a clinical entity was recognizable by community members. Most importantly, ‘feeling depressed’ was understood, but was not common to the lexicon of emotions. Instead, participants frequently endorsed excessive worry, grief and loss, and concern for family as the primary contributors to depressive moods. Key mood symptoms were excessive sadness and feelings of grief, irritability and anger. Cognitively, excessive rumination, homesickness and loneliness when away from their family and country, and suicidality were also frequently expressed emotional elements of depressive affect. However, the most consistent symptom of depressive affect among Indigenous men was the feeling of a weakened spirit, frequent and heavy use of alcohol, marijuana and other substances and the conduct of acts of spontaneous violence. Surprisingly, there was little mention or endorsement of feelings of excessive guilt, hopelessness, anhedonia, or evidence of symptoms of anxiety.

### Review of existing inventories

Potential depression screening instruments were then identified through a review of the existing literature focusing on previous epidemiological or clinical research that involved screening Indigenous community members or psychiatric patients for depressive symptoms. Identified measures were then interrogated by research staff according to a key set of pre-defined criteria (Table 1). Four screening instruments, Centre for Epidemiological Studies Depression Scale [CES-D] [23]; Kessler Psychological Distress Scale [shortened 6-item form - K6] [24]; PHQ-9 [8] and the Major Depression Inventory [MDI] [25], met these initial criteria and were therefore considered suitable to be included in a structured negotiated assessment of cultural equivalence.

**Table 1:** A-priori criteria for determining the choice of depression screening instruments for adaptation in Aboriginal communities in Central Australia

1	Likely face validity within the target population
2	Minimal or no culturally inappropriate questions
3	The existence of response categories that were linguistically and conceptually adaptable across languages
4	Brevity
5	Could be self-completed, as well as facilitated by an interviewer with little or no training
6	Had been used in cross-cultural studies previously
7	Possessed robust psychometric properties
8	Had been used in both psychiatric and community samples
9	Had been used in patients with medical co-morbidity
10	That there was concurrence/coherence with the primary findings from the qualitative fieldwork.

### Focus groups

Focus groups of been two to four translators, elders or bi-lingual key informants were then established in five distinct local Aboriginal language groups: Arrernte, Pitjantjarjara, Anmatyerr, Warlpiri, and Pintupi/Luritja (a combined Western Desert dialect of two key language groups), to support a negotiated assessment process. Focus group methods are widely used in cross-cultural psychiatric research, and are considered a critical component of multi-method approaches to enhancing the content validity of newly developed [or adapted] psychological instruments – they can assist with clarifying local idioms of distress, help maximise the conceptual coverage of proposed measurement items, enhance a holistic understanding of the construct under examination, and ensure the cultural appropriateness of various instruments, their items and response categories [26-28].

Guided by methods outlined by Vogt et al. [26], the focus groups were designed to:

1. validate, confirm, enhance [and where required, extend] the theoretical domains collated from the initial qualitative methods,

2. translate and back-translate developed items to ensure equivalence of adapted instruments; and

3. support the appropriate measurement of items [from existing standardised assessment instruments and/or culturally-specific additions] in terms of acceptability, semantics, recognised terminology and cross-language equivalence.

In order to achieve a broad representation of key community ‘targets’, a purposeful sampling technique was used to identify participants from not only Alice Springs but also remote communities representing the major local Aboriginal language. Potential bi-lingual experts relevant to these language groups were also identified through community, hospital, legal and health service networks, with a particular focus on individuals who had previously been involved in translating and/or research.

Initial contact was made by the principal investigator [AB] and/or the Indigenous Research Fellow [RM], the broad objectives of the study were outlined, and an overview of the proposed process of translation was discussed. An initial meeting was established for each distinct language group, where a more complete outline of the process was considered and those people interested in being part of a focus group were invited to provide informed consent.

### Adaption method

**Figure 1:**
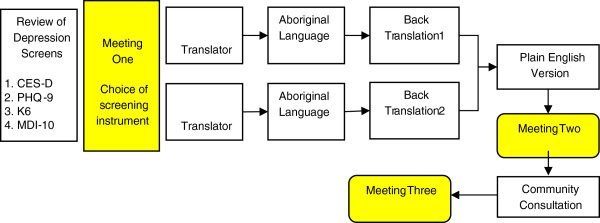
Outline of the translation and adaptation process for the MHM study.

Based on foundational work from Brislin [29] and practical guidelines outlined by Van de Vijver and Hambleton [30], a structured adaptation and translation process was developed (Figure 1). Each focus group participant was facilitated through the adaptation process with the aid of a ‘Plain English’ Translation and Adaptation Guide.

The first critical component of the Translation and Adaptation Guide was to determine the cross-cultural validity of existing instruments. This cultural assessment was undertaken jointly by the research team and bilingual experts/focus group participants at the first full meeting (Figure 1), and considered the equivalence of Content [*item relevance*]; Semantics [*that the questions held the same meaning across languages*]; Concept [*similarity of theoretical construct*]; and Technical features [*the appropriateness and method by which each question was asked*] [20] for each of the existing instruments. Based on the outcomes from this collaborative assessment, each focus group was then asked to identify the instrument, which they felt offered the most harmonious (and valid) approach. From the four acceptable instruments, each focus group independently chose the PHQ-9 as the most appropriate and easiest to translate.

A structured translation process outlined in Figure 1 was then implemented. In the first instance two bi-lingual experts from each language group were asked to translate the PHQ-9 instructions, questions and their response categories. Each translation was then discussed with the research team, clarity sought on meaning for difficult items, and problematic translations identified, discussed and amended (where necessary).

Translations were then recorded in the specific Aboriginal language using a digital recorder. The translations were then taken to another bi-lingual expert, who listened to the recordings, wrote the Aboriginal language version and then back-translated the instructions, items and responses into English. The back-translated versions were then reviewed in a second meeting by all of the focus group participants and the research team, to discuss (and where necessary reformulate and re-translate) incongruent questions. In particular, questions that had divergent English meanings between the two translators, or tapped seemingly incongruent underlying concepts were discussed openly.

Focus group participants were also able to take copies of the instrument back to their community, where discussions were held within their family networks about the study and the process of adaptation. This additional community consultation proved invaluable for a number of reasons. Not only did it contribute to a broader understanding of the linguistic nuances between language groups but also helped to explore the within-group acceptability of the adapted PHQ-9 according to demographic factors such as age and gender. Together these family group discussions were integral to the development of conceptual equivalence for several key domains of the existing screening instrument.

A final focus group meeting was then facilitated to re-visit the difficult items, to explore their meaning (in English) and identify potential ways to re-translate them in a manner that would achieve equivalence of meaning. On occasion, this would involve review of the approaches that other (different) language groups took to translate items. Re-worded items were then translated into language by the bilingual experts and back-translated until consensus and clarity was achieved. The consensus translations of each language group were then combined and discussed with all translators to ensure consistency across languages, and to reach agreement on a single ‘Aboriginal English’ [or plain English] version to be used in the field.

## Results

The translation and adaptation process required significant negotiation across all language groups, taking approximately six months to complete. While, there were several key conceptual difficulties exposed throughout the process, these related largely to the construction of hopelessness. Depressed mood was consistent and translatable in all languages. The individual PHQ-9 items are discussed below, with informative examples of the translation and adaptation process.

### Anhedonia

Item 1, relating to a lack of interest or enjoyment of usual activities, was able to be translated across all languages, but was strongly framed around local vernacular. As such it was an item requiring a search of linguistic rather than conceptual equivalence. This was largely framed around ‘slackness’ or ‘feeling slack’, ‘not wanting to do anything’. Initial back-translations from Luritja related anhedonia to a sense of tiredness and unhappiness, but negotiation was able to differentiate this from a lack of energy.

### Depressed mood

The centrality of the ‘spirit’ in the emotional and physical expression of depression was clear. In all languages, spirit, and its perceived wellbeing was the most appropriate, conceptually equivalent expression for translating depressed mood (Table 2). Depression, as a construct, had several distinct equivalents that served to package the elements of what translators considered to be ‘depressed mood’ together. Rather than attempting a direct translation, initial discussions with Arrernte speakers focused on a broader question of feelings, “*How do your feelings feel?*” Or “*How do you feel deep down inside?*” These questions were considered appropriate by bi-lingual experts because they felt it would be unusual to ask someone about their feelings without a transactional discussion of the context or circumstances in which that emotion was constructed and experienced.

**Table 2:** Initial and consensus translations of PHQ-9 Question 2 - Depressed mood

“***Over the last 2 weeks, how often have you been bothered by feeling down, depressed, or hopeless?”***			
**Language**	**Back translation 1**	**Back translation 2**	**Within language consensus**
**Arrernte**	Are you feeling down, depressed?	How do your feelings feel?	Have you been feeling sad, down, depressed, no good?
**Pitjantjatjara**	Are you always unhappy, lonely, and feeling sad all the time	Is your spirit sick, sad or homesick?	Have you been feeling unhappy, lonely, sad or your spirit is sick?
**Anmatyerre**	Is your spirit feeling no good?	Do you feel no good, nothing makes you feel better?	Have you felt depressed, no good, your spirit feels no good?
**Warlpiri**	Do you feel down, sorry for yourself?	Have you been feeling sad and sorry for yourself?	Have you been feeling really sad about yourself?
**Luritja**	Are you feeling sad inside?	Do you have an overwhelming sadness inside?	Have you been feeling sad inside?
**Final adaptation**	*“Have you been feeling unhappy, depressed, or really no good, that your spirit was sad?”*		

In many respects Arrernte language speakers had a direct translation for depression, which represented the emotion as well as the constellation of feelings and behaviours that aligned with a depressive syndrome. These emotions included anger, a deep hurting inside, ‘big sadness’, loneliness, lethargy and weak or low spirit. As such depression was equivalent to the Pitjantjatjara construction of a weakened or sick spirit (*kurunpa*), which was also confirmed by the Pitjantjatjara and Luritja bilingual experts. The Arrernte term *kurunpa* was also directly equivalent to that used in Anmatyerre. Warlpiri translators also proffered an equivalent, but slightly different term, framed more in line with Pitjantjatjara term for homesickness [*watjilpa*]. *Watjilpa* did, however, represent the same emotional phenomenon. These terms were considered a reflection of a cluster of emotions and behaviours that occurred as a result of depressed mood.

Given the conceptual equivalence of meaning but slightly different linguistic expressions, the final, cross-language consensus process included key words from each language to ensure translatability of *both* words and meaning. The final question read as ‘Have you been feeling unhappy, depressed, really no good, that your spirit was sad?’

The original PHQ-9 also housed a sub-component of hopelessness within the depressed mood question, ‘*In the last two weeks, how often have you been feeling down, depressed, or hopeless?*’ The construction and measurement of hopelessness provided significant conceptual, linguistic and translational difficulties for bilingual experts. All translators felt that the overarching equivalent was the constellation of depressive feelings and therefore left hopelessness out of the PHQ-9 adaptation.

### Vegetative symptoms including sleep disturbances, appetite, lethargy, and psychomotor changes

There was also significant concern with the loading of opposite or multiple questions within the one sentence, as seen in question five, on appetite changes ‘poor appetite or overeating’, and question eight, on psychomotor changes ‘Moving or speaking slowly that other people could notice’. Or the opposite – ‘Being so fidgety or restless that you have been moving around a lot more than usual?’.

Whilst translators felt that it was possible to translate these questions linguistically, it made little sense to do so, as it was possible that the questions would confuse and potentially annoy some interviewees. The decision was made to separate these questions into two distinct sub-elements, which were then translated and back-translated as separate items.

Appetite required consideration of the contextual realities of Aboriginal people in the face of significant socioeconomic hardships. The translation of poor appetite was largely as ‘not eating’, rather than disinterest in consumption. Each language group suggested that it was not unusual for people to go without food for many days at a time, largely as a consequence of poverty, rather than appetite per se. Further, ‘not hungry’ was not felt to be specific enough to identify people with depressive disorder. As a result, context was added within the newly created question 5a and 5b:

5a:‘Have you not felt like eating much even when there was food around?’

5b:‘Have you been eating too much food?’

This was also discussed as a possible solution to item 3 ‘Trouble falling or staying asleep, or sleeping too much’. However, four of the five language groups were comfortable with a broader approach to sleep disturbance suggesting either ‘difficulty sleeping at night, or trouble with sleeping’ as a broader category.

### Self-reproach and negative cognition

The translation of negative self-perceptions faced several challenges. As was the case with discussions around depressed mood, translations focused on both the feelings inherent in negative thought, and behaviour as a response to those feelings, as can be seen with the Warlpiri back-translations (Table 3).

**Table 3:** Initial and consensus translations of PHQ-9 Question 6 – Self-Reproach

***“Over the last 2 weeks, how often have you been bothered by feeling bad about yourself- or that you are a failure or you have let yourself or your family down?”***			
**Language**	**Back translation 1**	**Back translation 2**	**Within language consensus**
**Arrernte**	I can’t do it, I have feelings that are no-good	How are your feelings? Are you thinking hard about your feelings?	Have you been feeling useless, no good, that you can’t do anything?
**Pitjantjatjara**	Do you think that you are a bad person and you have ruined everything for you and your family?	Do you think that you are a no good person?	Have you been feeling you were a bad person and you ruin everything for you and your family?
**Anmatyerre**	I feel sick physically or with emotional sadness, loneliness and depression.	Are you feeling sad because you are a bad person?	Have you been feeling bad about yourself, sad and depressed, that you are no good?
**Warlpiri**	I feel bad about myself and about my family because I let them down.	I feel really tired and bad. I want to walk away from people and family.	Do you feel bad about yourself and about your family because you let them down?
**Luritja**	Has your spirit been feeling no good?	How is your thinking? Are you thinking bad things?	Has your spirit been feeling no-good?
**Final adaptation**	*“Have you been feeling bad about yourself, that you are useless, no good, that you have let your family down?”*		

Lay descriptions of people who had let their family down included ‘rubbish’, ‘no good’, or ‘useless’ which also framed the adapted consensus wording. Luritja and Anmatyerre language speakers framed negative self-perception around sadness, and loneliness as a consequence of feeling bad about one’s self. Given the incongruence with the original PHQ-9 question, review of other language translations assisted with comprehension and allowed the final version to cover feelings of inadequacy.

### Difficulty concentrating

Poor concentration was initially difficult to adapt, and the use of watching television or reading newspapers (as in the original PHQ-9) was not considered an appropriate cue. ‘Thinking straight’ or ‘thinking clearly’ was the most appropriate equivalent. The Pitjantjatjara translations focused on the application of thinking in the context of Aboriginal life, posing that difficulty concentrating would manifest as an inability to remember or learn new stories.

### Suicidal ideation

Suicidal ideation was commonly agreed to be an important sign of depressed mood and negative emotions across all focus groups. Whilst the question on self-harm and suicide was easily translatable by all language speakers, there was some concern with the appropriateness of asking people this question, and about what we would do if someone answered that they were feeling this way. External consultation with communities found that while they were also concerned, they felt that it was an important question to include.

### Response categories and instructions

The appropriateness and timing issues inherent within the instructions and response categories were considered by focus group participants and found to be readily translatable. The ‘preceding two weeks’ and grading severity of symptoms within a time-based framework was possible (and deemed appropriate) across all languages.

Rather than simply transposing western labels of symptoms, this extensive process led to a new, adapted measure (Table 4) which was conceptually equivalent to the original PHQ-9. While researchers facilitated this process, it was clear that the community consultation to ensure both acceptability and validity across Indigenous Australian communities were key. This was achieved through a highly engaged consultation process involving the focus groups representing five different language groups together with various family and community discussions.

**Table 4:** Final consensus questions- adapted PHQ-9

**Questions**		**None**	**A little bit**	**Most of the time**	**All of the time**
**In the last two weeks, how often have you been feeling the following:**					
1	Have you been feeling slack, not wanted to do anything?	0	1	2	3
2	Have you been feeling unhappy, depressed, really no good, that your spirit was sad?	0	1	2	3
3	Have you found it hard to sleep at night, or had other problems with sleeping?	0	1	2	3
4	Have you felt tired or weak, that you have no energy?	0	1	2	3
5a^$^	Have you not felt like eating much even when there was food around?	0	1	2	3
5b^$^	Have you been eating too much food?	0	1	2	3
6	Have you been feeling bad about yourself, that you are useless, no good, that you have let your family down?	0	1	2	3
7	Have you felt like you can’t think straight or clearly, its hard to learn new things or concentrate?	0	1	2	3
8a^$^	Have you been talking slowly or moving around really slow?	0	1	2	3
8b^$^	Have you felt that you can’t sit still; you keep moving around too much?	0	1	2	3
9	Have you been thinking about hurting yourself or killing yourself?	0	1	2	3
		**Total score (0–27)**			

^$^Note: Scores for depressive symptoms - record only the highest in each of these sub-questions.

## Discussion

Despite the high burden of mental illness and social and emotional wellbeing issues within Indigenous Australian communities, the identification of depression and related factors poses many challenges - linguistically, conceptually and practically. Unfortunately, the need to better understand and explore the prevalence, patterns and consequences of conditions such as depression, has not up until now, been met with the development of robust, culturally aligned and validated population screening instruments [3]. This current study has now begun this important work by both translating and adapting the PHQ-9 for use across multiple Indigenous Australian language groups.

Previous attempts to assess this burden of depression for Indigenous Australians have been fraught with complexity. The use of standard measures presumes a universality of definition and understanding which is inappropriate. Australia’s National Health and Medical Research Council [18, p. 3] cautions researchers about the need to critically consider cultural differences when measuring and comparing Indigenous and non-Indigenous Australian health outcomes. Labeling Indigenous people with western diagnostic classifications, without assessment of their equivalence, relevance, acceptability or utility serves little purpose if such labels are devoid of the context and realities of Indigenous people’s lives, their many languages, or the way in which Aboriginal people experience and express psychological distress [19].

Clearly, the construction of depression must be understood within the social, moral, and cultural context of the population of interest [31]. When considering the development of psychological screening instruments within a defined cultural group, one must also decide how far to go to develop local, culturally specific questionnaires [32,33], which, whilst valid on a local scale, cannot be compared to external populations or to other well validated and widely used instruments. One possible solution is to work towards an extensive process of adaptation of robust, validated instruments. Despite the existence of guidelines and examples of facilitated adaptation and translation processes for these types of psychometric instruments [34], adaptation with Indigenous communities has not routinely occurred. This is not surprising given the complexity of the task.

One of the primary reasons for this complexity is the degree of cultural diversity within the Australian Indigenous population. The need to accommodate as much as possible the many different language/cultural groupings makes it inherently more complicated than the simple process of adapting a screening and diagnostic instrument from one culture into another. Furthermore, we found that even the available guidelines made little mention of the additional requirements our bi-lingual experts considered essential – community and family endorsement of their translation and conceptual alignment. These complexities have hamstrung the necessary focus on and measurement of the significant impact of psychological factors on the experience of health and wellness within Australian Indigenous communities.

Despite these challenges, the extensive process of translation and adaptation in this study has demonstrated that within the context of Indigenous Australian communities, across multiple language groups, where English is often a third or fourth language, conceptual and linguistic equivalence of psychological constructs can be negotiated. As an important start to this work, we were fortunate to be able to conduct extensive qualitative examination of distress and emotional expressions within the target population. Five focus groups became the ‘expert panel’ throughout the structured adaptation. Each of these focus groups independently chose the PHQ-9 instrument to undergo adaptation, because of it brevity, use of simple English, lack of inappropriate questions and face validity.

The PHQ-9 [8] was established as a short form, comprises nine depression-specific questions which directly relate to Diagnostic and Statistical Manual of Mental Disorder IV [35] criteria for diagnosing depression, serving as both a case-finding diagnostic instrument as well as grading depression severity [8]. In addition to being considered acceptable for adaptation, it is also considered to be appropriate for use in a primary health care setting and is widely used to both diagnose and establish prevalence estimates of depression [4]. Previous studies have demonstrated that the PHQ-9 works comparably across multi-ethnic populations in the United States [36,37] and has been translated into Spanish, Chinese, Thai and Swahili [37-39]. The PHQ-9 has also been adapted for use in a small sample of Aboriginal primary care patients with coronary heart disease [7].

Most of the difficulties encountered by this study in adapting the PHQ-9 for use in Indigenous Australian communities related to several interlocking issues. First, while depression and distress were considered important psychosocial factors within Aboriginal life and validated instruments offer useful starting points for their measurement in epidemiological research, there remained important expressions within standardised PHQ-9 that are inherently culturally bound. These expressions required extensive translation of meaning to commence adaptation. Second, the search for conceptual equivalence was at times prohibitively slow, particularly around the construction of ‘hopelessness’. This proved even more complex, in the recognition that emotional inventories or lists of questions are usually devoid of context and often separated from their broader social meaning. In a group that sees little separation between the mental and physical elements of their lives, or between the social, biological and psychological construction of illness, asking questions about emotions without contextual cues, space for open discussion or reason were difficult to reconcile. At all times, however, the research team was blessed with the patience and unparalleled skills of Aboriginal bi-lingual experts, who took a great personal and professional interest in the aims of this work.

Each language group brought particular linguistic and conceptual skills to bear on the process. For example, the Warlpiri group was able to provide the first insight into the potential clarification of an equivalent method of asking respondents about hopelessness, which provided the subsequent clues to the Arrernte group to complete the translation process. This linguistic cross-fertilisation proved invaluable, as at points of impasse, one group was able to unlock seemingly intractable and frustrating difficulties. Beyond the conceptual struggles, the practicalities of asking many questions within one single item, particularly if it involves seeking opposite ends of a continuum, was considered problematic.

Finally, whilst we were able to develop translated versions of each instrument across five languages, these were agglomerated into a single, simplified English version built on the strengths of each, that can be used across heterogeneous settings, and provide the ‘code’ and specific *conceptually* equivalent words that could be used by translators across languages. We contend that both the process by which the PHQ-9 was adapted, and the instrument itself, should provide an exemplar to support primary care psychological assessment and epidemiological fieldwork.

Now that the translation and adaption process is complete, there is a need to undertake psychometric testing [34]. A validation study against a gold standard in order to assess its potential as an instrument for measuring the burden of disease across Indigenous Australian populations is required. In addition, an assessment of the score level attributes including the optimal cut-off score which will bring the number of false positives and false negatives closest together, thereby off-setting any potential sources of error must needs to be undertaken. Further research is also required to assess the reliability of this newly adapted PHQ-9.

## Conclusion

This paper outlines the steps taken to adapt the PHQ-9 for use across Indigenous Australian communities. Avoiding the temptation to directly translate western concepts into locally acceptable labels, this extensive process of both translation and adaptation has demonstrated that within the context of Aboriginal communities, across multiple language groups, where English is often a third or fourth language, conceptual and linguistic equivalence of psychological constructs can be negotiated. While the key significance of this study was the development of an instrument which has the potential to measure the burden of depression across Aboriginal communities, this paper also describes a process which could be used to adapt screening instruments for other psychiatric disorders.

### RATS Guidelines

The qualitative study described in this manuscript complies with the qualitative research review guidelines – RATS.

## Endnote

^a^For more extensive guidance see http://www.clc.org.au/articles/cat/aboriginal-languages-of-central-australia/; or visit http://iadpress.com.

## Abbreviations

PHQ-9: Primary health questionnaire 9.

## Competing interests

The authors declare that they have no competing interests.

## Authors’ contributions

AB – Designed the study, contributed to the data collection, undertook the data analysis and drafted the paper. RM – Contributed to the data collection, assisted with the data analysis and reviewed the paper. KR – Contributed to the design of the study, assisted with the data analysis and reviewed the paper. TS – Assisted with data analysis and drafting of the paper. CD – Assisted with data analysis and drafting of the paper. KOD - Contributed to the design of the study and reviewed the paper. All authors read and approved the final manuscript.

## Pre-publication history

The pre-publication history for this paper can be accessed here:

http://www.biomedcentral.com/1471-244X/13/271/prepub
